# Safety assessment of charcoal usage and effects of common charcoal ignition aiders on combustion indices

**DOI:** 10.1038/s41598-022-21059-w

**Published:** 2022-10-08

**Authors:** A. O. Ajayeoba, M. O. Fajobi, K. A. Adebiyi, W. A. Raheem, S. O. Oladapo, M. D. Olayinka

**Affiliations:** 1 Department of Mechanical Engineering, Ladoke Akintola University Technology of Ogbomoso, Ogbomoso, Nigeria; 2 Department of Mechanical Engineering, University of Ilorin, Ilorin, Nigeria; 3 Open and Distance Learning Centre, Ladoke Akintola University of Technology, Ogbomoso, Nigeria; 4 Department of Systems Engineering, University of Lagos, Lagos, Nigeria; 5 Department of Mechanical Engineering, Olusegun Agagu University of Science and Technology, Okitipupa, Nigeria

**Keywords:** Engineering, Mechanical engineering

## Abstract

Charcoal is a popular form of biofuel embraced for domestic and industrial purposes. However, the use of Charcoal has some associated challenges, such as the required charcoal pot and setting it into the fire at first by using Charcoal-Ignition-Aiders (CIA) (e.g. discarded paper, nylon, rubber, plastics, petrol, the residue of processed palm oil, maise cob, wood, and kerosene). Coupled with the chemical properties of Charcoal, the resulting gases from CIA are capable of polluting the environment with perceived Adverse-Health-Implications (AHI) on the ecosystem. Therefore, this study conducted a safety assessment of charcoal biofuel usage and the effects of common CIA on combustion indices. This study followed standard methods and the use of peculiar equipment. This study established that Charcoal is commonly used in the studied area because it is cheap, readily available and requires less technical know-how. Considering the combustion indices, using paper as a CIA generated the lowest carbon monoxide (CO) value, 28.1 ppm, with 3,434.54 ppm volatile organic compound, VOC. Compared with the ACGIH standard permissible exposure level of ≤ 30 ppm, the paper gave a lesser CO value of 28.10 ppm among all the CIA. At the same time, all the CIA recorded higher VOC compared with EPA standard permissible exposure level of ≤ 15 ppm. ANOVA analysis conducted on the socio-demographic profile of the respondents, cooking attributes of the respondents, and use of charcoal pot types by the respondents in Zone 1, Zone 2, and Zone 3 gave p-values of 0.032, 0.028, and 0.039, respectively. These imply significant differences within the zones in each of the indices. The average energy content reported for charcoals sourced from oak trees, afara, obeche, mahogany, and iroko woods is 3,2149 kJ/kg compared to the lower ones. Therefore, this study recommended using these charcoals alongside discarded paper as CIA because they are a better combination to reduce AHI.

## Introduction

The energy crisis emanating from the rapid increase in global energy demand, instability observed in the global fossil fuel marketing, continued greenhouse gas emissions to the atmosphere and environment, and extinction of fossil fuels is an emerging but unpalatable global phenomenon^1–3^. These factors informed the need for active heterogeneous exploration of available renewable energy resources to salvage the situation^1–3^. Majorly, renewable energy is such that it always replenishes even after use, thereby making them inexhaustible^4,5^. The conversion processes for deriving biomass energy include direct combustion and anaerobic digestion. Others are pyrolysis or gasification, hydrogenation or fermentation, hydrolysis, and briquetting, amongst others^6^. In this context, the latter's product is preferable for direct combustion in households^7^. Biomass includes the remnants of food, farm residues, disposed of refuse, and discarded materials that can be processed and later converted to other suitable forms, such as Charcoal. According to Mariusz et al*.*^8^, and IEA^9^, biomass constitutes 10% of the global world’s primary energy supply, substantiating the importance of renewable energy. Also, Eniola et al.^10^, inferred that more than 2 million people domestically implement Charcoal, wood, and other agricultural residues as the primary fuel for cooking, space heating, and lighting purposes, resulting in significant health, economic and environmental implications. In Nigeria, 72% of the population still depends on the use of Charcoal and fuelwood^11^. Although, other common fuels such as kerosene and liquefied natural gas for home use are still slightly in use.

Previous research revealed that apart from the price of kerosene and cooking gas which has escalated because of the removal of fuel subsidy, cooking gas can only be procured by those ready to pay the high price, thus making kerosene a scarce commodity in Nigeria^12^. Attributed characteristics such as availability, cheapness, abundance, ease of use, and improved calorific value have made Charcoal a popular form of fuel for industrial and domestic purposes. Charcoal is mostly pure carbon known as char produced by the cooking of woods in a low oxygen environment which takes some days^13^. The cooking is to burn some volatile compounds like water, methane, hydrogen, and tar. But when produced commercially, this burning occurs in a large hole, concrete, bricks, and steel silos with little oxygen. Stopping the wood-cooking halfway is necessary to avoid burning to ashes^14–16^. The heat generated by Charcoal is about 2700 °C (4890 °F), suggesting its suitability as metallurgical fuel because it burns at intense temperatures. The use of Charcoal as fuel for household use requires that a charcoal pot (stove) serves as a conveying medium in which the charcoal pieces before applying fire^17,18^. As suitable as a charcoal pot may be, it still has some peculiar challenges associated with its use, such as handling the pot, which body cuts and burns may accompany.

Others are foot injuries, respiratory hazards, and releasing harmful pollutants (especially when burnt in inefficient stoves), which harm man, the environment, and the ozone layer^19–21^. Furthermore, fuelwood is still important in African and Asian countries, as it is in use mostly in rural and many cities^22,23^. Thus, its use is associated with dirty-burning fuels, which results in some health risks and hazards from the polluted air^22,23^. Globally, issues associated with the choice of household energy and transition are crucial, especially from assessment and policy viewpoints^24^. Thus, advocacy and concerted efforts have encouraged households to make substitutions that will result in more efficient energy use and drastically reduced adverse environmental, social, and health effects. Achieving this demands research and analysis of the factors responsible for choosing Charcoal as fuel, ignition timing, ignition aiders, combustion properties, and safety assessments of using Charcoal domestically. The use of Charcoal, though, burns with neater and cleaner flames compared with fuelwood and kerosene, comes with many risks and potential hazards. Lightening up the Charcoal for ignition requires several ignition aiders that, in turn, constitute offensive emissions of harmful gases such as nitrogen oxides and sulphur. These emissions vary across regions, states, provinces, and nations and the differences are affected by factors such as ventilation, population density, fuel types, and anthropogenic activities. This study assesses the suitability of charcoal fuel and the accompanying ignition aiders commonly used in setting up Charcoal into the fire, considering their combustion indices.

The common ignition aiders are mostly; discarded paper, nylon, rubber, plastics, petrol, the residue of processed palm oil (locally known as *oguso* in south-western Nigeria), maise cob, wood, and kerosene. The presentation of scientific names of these ignition aiders is in the methodology section of this study. Asides from the chemical characteristics of Charcoal itself, the resulting gases from any of these highlighted ignition aiders is/are capable of standing as pollutants to the environment and have adverse health implications on the ecosystem. The menace is not only experienced in Nigeria alone but in the world at large, especially in countries where the use of Charcoal for heat generation is prominent. On the other hand, these informed the need for this study to establish the forms of gases emitted from these ignition aiders and to evaluate their conformity to standards and acceptable levels. Therefore, the objective of this study is to assess the safety level of the use of charcoal fuel as well as investigate the effects of common ignition aiders on combustion indices.

## Methodology

This study area considered was Ogbomoso (8°8′31.79ʺN, 4°1′42.67ʺE), Southwestern Nigeria. The study area has three zones: Ladoke Akintola University of Technology area [Zone One (Z_1_)], Ogbomoso North Local Government [Zone Two (Z_2_)], and Ogbomoso South Local Government [Zone Three (Z_3_)] areas. Zone One (Z_1_) is a student-residential area, and student-related activities characterise the zone. Zone One (Z_2_) is an off-campus area occupied mainly by the indigenes, and the major commercial activities of the location are marketing and transportation. Zone One (Z_3_) is also indigene-occupied but located at the extreme of the study area, and farming activities are the peculiarities. The study used a standard table of sample size, confidence levels, and confidence intervals for stratified and random samples to determine and select sample sizes. That is, the Charcoal users whose responses were statistically significant to generalise the opinions of all charcoal users in the study area) from all the three zones of the study area^25^.

**Figure 1:**
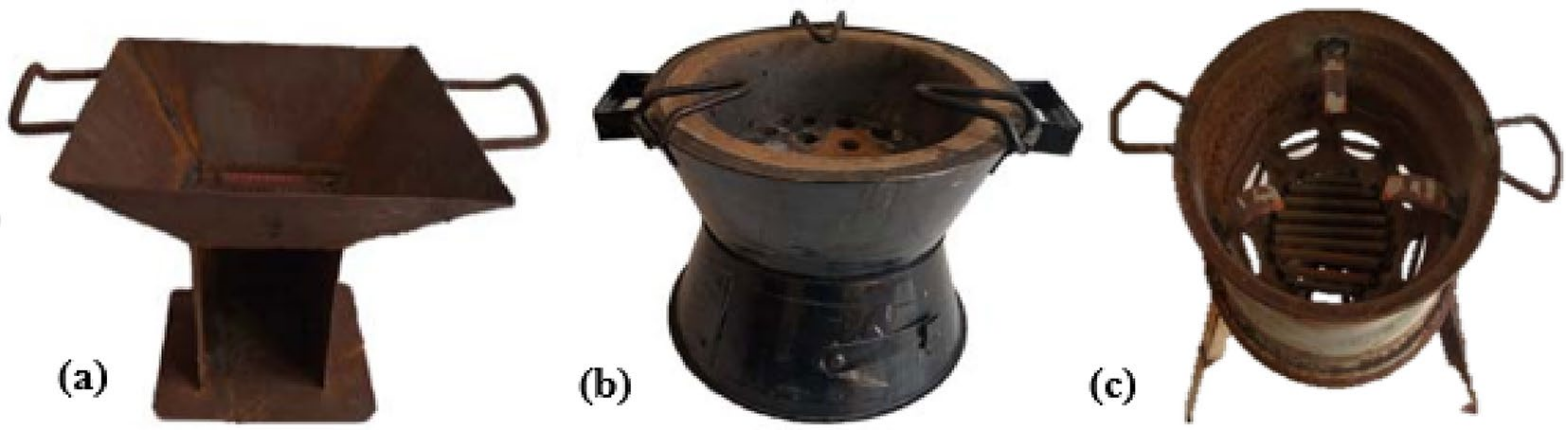
(**a**) Open air inlet. (**b**) Closed air inlet. (**c**) Open base.

Six hundred (600) respondents were statistically determined and sampled for safety assessment on the use of three common charcoal pots (Fig. 1). The design and administration of the structured questionnaire followed the standards of European, Spanish, and Latin American surveys, recommendations of documentary research, and exhaustive reviews of previous works on occupational health and safety^26^. Strict adherence to the guidelines and regulations was necessary to avoid ambiguity and to enhance clarity and simplicity. In addition, the authors communicated the purpose of the research and pertinent information to the respondents. Meanwhile, before questionnaire administration, the respondents gave informed consent. Finally, the authors administered the questionnaire to collect the respondents' demographic information such as age, sex, marital status, and ethnicity. Evaluation of various energy resources used and cooking attributes of the respondents, including their safety preparedness in terms of dress mode while using charcoal pot, as well as the reason for preferring Charcoal compared to other energy resources. Also, this study assessed hazards and safety practices, the stresses involved in getting Charcoal, burning, and drying it when wet, and events and circumstances surrounding its use. The study investigated nine types of common ignition aiders in the study area (i.e., paper (printed-on type), nylon, rubber, plastics, petrol, *oguso* (the residue of processed palm oil), dried-maise cob, wood, and kerosene). To establish their suitability for use and effects on the health implications of the respondents. The Charcoal used was purchased from a local market within the study area. Experimentation was at an average ambient temperature of 28.3 °C and average relative humidity of 56.2% with a GPS temperature/humidity data logger (Model No: GPS-6).

The study used a bomb calorimeter (Model No: CAL3K) to determine the calorific value of the purchased Charcoal using the ASTM D5865-04^27^. Then, a 10 kg quantity by weight of the purchased Charcoal was measured using a weighing scale (Model No: Models no: Z051599 of 20 kg maximum capacity). Also, the study used the same weighing scale to measure 2 kg of each ignition aider. The authors firstly loaded the measured Charcoal into a metallic charcoal pot (locally made) and then added the ignition aider in the middle. Then, the authors ignited the aider. The study used a portable handheld gas analyser (Aeroqual Model: Series 500) to analyse the Carbon-monoxide (CO), Nitrogen dioxide (${\mathrm{NO}}_{2}$), Ozone (O_3_), and Volatile Organic Compounds (VOCs) contents of the emitted gases. The analyses were after two minutes; the Charcoal fully caught fire. The position of the gas analyser was at a distance of 1 m from the emission source for data collection. All experiments and emission analyses were under ambient conditions, i.e., outdoors (in the open air). The gas analyser was held for at least 2 to 3 min (in line with the manufacturer’s guide) in the direction of the emission source while data collection and analysis were on. Measurements were taken in triplicates, each from three different directions (with the same distance to the emission source) away from the emission source. The averages of the recorded data have been reported in this study. The highlighted procedures for experimentation were followed for each ignition aider, and data were recorded, respectively. Collated data were presented on an excel spreadsheet in preparation for statistical analysis (descriptive, charts, and ANOVA) on a statistical package for social sciences, SPSS 23.0 version.

The current study also analysed each ignition aider to determine the elemental compositions; carbon, hydrogen, nitrogen, sulphur and oxygen. Analyses of each ignition aider's calorific values were with Parr 6200 oxygen bomb calorimeter (Model No: A1290DDEE). The process followed the ASTM D5865-0446 standard. ASTM D4239-11^28^ standard was adopted in completely combusting a 2 g quantity of each solid ignition aider. LECO-CHN628 Handheld Analyser (Model No: 622-000-000) was used to analyse carbon, hydrogen and nitrogen samples. LECOS-144DR Sulphur determinator (Model No: 606-0000-300, SN-477) was used to analyse the sulphur contents. ASTM D187-18^29^ Standard and ASTM D6045^30^ Standard were used for analysing the liquid ignition aiders, i.e., kerosene and petrol, respectively. The percentage of oxygen was obtained by a positive difference between 100 and the sum of the carbon, hydrogen, nitrogen, and sulphur contents.

## Results and discussion

### Socio-demographic profile of respondents

The population sampled was female predominant because of the general belief that females are primarily involved in cooking and other house chores than males. The result suggests a higher percentage recorded for females across the three zones of the study area, i.e., zones 1, 2, and 3 were 60, 75, and 84%, respectively, while the percentage of males was 40, 25, and 16% respectively. In all the zones, respondents in the age range of 20 to 44 years were prevalent with 40%. In contrast, 45 and above recorded a cumulative of 35.8%. Across all age categories, zones 1 and 2 respondents were of a lesser age bracket than zone 3. This result might be because zones 1 and 2 are students-dominated areas (see Table 1). Considering the marital status of the respondents in all three zones, the percentage of single, married, and widows are 33.7, 59, and 7.3%, respectively. The representation of married being the highest of it all suggests the involvement and huge responsibility posed before females, especially the married, such that preparation of food for their husband and children lies on them.

**Table 1:** Socio-demographic profile of respondents.

Variables		Zone 1	Zone 2	Zone 3	Total (%)
**Sex**	Male	80 (40.0)	50 (25.0)	32 (16.0)	162 (27.0)
	Female	120 (60.0)	150 (75.0)	168 (84.0)	438 (73.0)
**Age group**	< 20 years	70 (35.0)	60 (30.0)	15 (7.5)	145 (24.2)
	20–44 years	98 (49.0)	80 (40.0)	62 (31.0)	240 (40.0)
	45–70 years	25 (12.5)	48 (24.0)	111 (55.5)	184 (30.7)
	70 years and above	7 (3.5)	12 (6.0)	12 (6.0)	31 (5.1)
**Marital status**	Single	126 (63.0)	82 (41.0)	20 (10.0)	202 (33.7)
	Married	70 (35.0)	110 (55.0)	148 (74.0)	354 (59.0)
	Widowed	4 (2.0)	8 (4.0)	32 (16.0)	44 (7.3)
**Ethnicity**	Yoruba	170 (85.0)	180 (90.0)	188 (94.0)	538 (89.7)
	Hausa	10 (5.0)	15 (7.5)	8 (4.0)	33 (5.5)
	Igbo	20 (10.0)	5 (2.5)	4 (2.0)	29 (4.8)

Bracketed figures represent the percentages of each figure (frequencies) beside them.

In contrast, the foodstuff exploration lies in their husbands' hands. These results were consistent with those obtained for the ethnicity distribution of the current study, where Yoruba topped the ranking with 89.7%, followed by Hausa, 5.5%, and the least by the Igbo tribe, 4.8%. The study area is in the South West of Nigeria, where the Yoruba tribe is prevalent, and they believe that the cooking of the food is exclusively for the female. This notion further substantiates the more significant percentage recorded in Table 1 for females’ (73%) involvement in cooking and other house chores compared to the males' (27%).

### Evaluation of energy resources and cooking attributes of respondents

**Figure 2:**
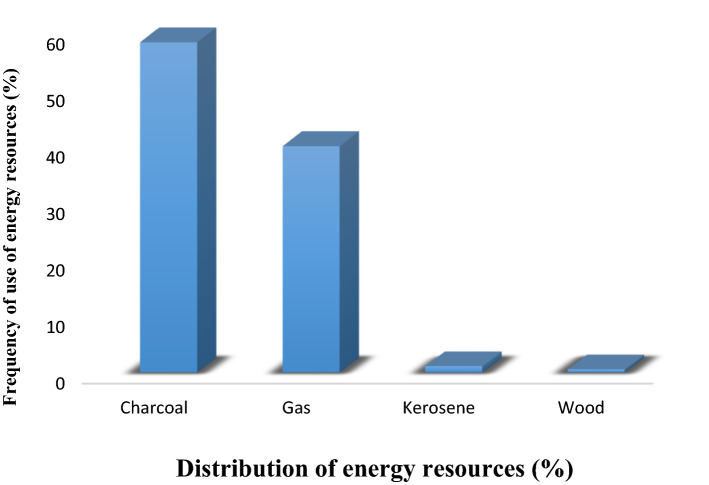
Distribution of energy resources (%).

This section evaluates the various forms of energy used for cooking in the study area and consequently explores the attributes of the respondents. From Fig. 2, the respondents' familiar and preferred energy resource for cooking is Charcoal, 58.33%, followed by gas, 40.01%. Using kerosene and wood was sparingly and sometimes a backup with 1.11 and 0.55%, respectively. The common reason for the choice of resources was because the cost of obtaining resources such as Charcoal is least expensive, stress-free (second to gas), and relatively fast compared to others. The implication is evident from the percentages obtained for least expensiveness (70%), stress-free (31.3%), and fastness 41.7%, Table 2. Another reason to corroborate this claim is that some opt for using Charcoal if there is no sufficient fund to purchase other forms of energy such as kerosene and gas, thus making them multiple users at some points. This reason is widely acclaimed, especially in the areas close to the students' areas, such as zone 1 and zone 2, whereby when students go financially broke, they settle for Charcoal and kerosene to support their daily lives. However, most users do not often regard the mode of dressing as necessary when using charcoal pot except for women who engage in sales of snacks by roadsides who wear aprons most times. Percentage respondents who put on an apron, native wear, English wear, and others are 4.7, 20, 33.3, and 42%, respectively. Through personal interactions and close examination, most of the respondents in and close to the students’ areas (zones 1 and 2) put on a nonchalant attitude toward the appropriateness of the dressing code when using Charcoal pot. However, in some cases, they only care to be dressed smartly to avoid careless contact with hazards. Therefore, in an attempt to key into standard safety practice, it is reasonable for the user of the Charcoal pot to wear appropriate clothing when using the Charcoal pot in preparedness to get rid of associated hazards, thus, averting any form of domestic accident.

**Table 2:** Cooking attributes of respondents.

Reasons for preference	Charcoal	Kerosene	Gas	Wood
Less expensive	420 (70)	38 (6.3)	120 (20)	10 (1.7)
Stress-free	184 (31.3)	40 (6.8)	354 (60.2)	10 (1.7)
Fastness	250 (41.7)	23 (3.8)	300 (50)	15 (2.5)
Others (kids handling, neatness, etc.)	210 (35)	20 (3.3)	274 (45.7)	5 (0.8)
**Multiple users**	350	50	400	20
**Single users**	264 (44)	27 (4.5)	299 (49.8)	10 (1.7)

Mode of dressing	Apron	Native	English	Others
	28 (4.7)	120 (20)	200 (33.3)	252 (42)

Bracketed figures represent the percentages of each figure (frequencies) beside them.

### Safety assessment of charcoal pot in the study area

Presented in Table 3 is the result of the assessment of safety parameters related to the use of charcoal pots, in terms of types, frequency of accidents occurrences, and probable reasons for accident occurrence with the evaluation of recommendation of the use of Charcoal as an energy resource. Of all the types of charcoal pot in consideration, the closed air inlet appears safest, although with less acceptability, 6.7%, probably because it is the most expensive and scarcely found. The most common type and popularly used is the open base which recorded 62.2%. However, it is the most vulnerable to accidents. Because when the wind blows, it spreads the remains of Charcoal deposited at the base, which could be hot and lead to burns for the users. Sometimes air can blow it to neighbouring flammable materials; this could lead to fire outbreaks resulting in loss of life and properties. The percentage of open-air-inlet charcoal pot type users is 31.1. Hwangdee et al*.*^19^, in a related study, established that using charcoal pots could be associated with varying degrees of accidents. The finding is consistent with the results obtained in the current study, where 74.5% of the respondents stressed that it is true that accidents occur primarily due to mishandling or by the wind. But 25.5% of the respondents agreed that a few occur due to carelessness. A more significant number of respondents, 60%, recommend using Charcoal. Still, it becomes necessary that users must be patient and willing to make sacrifices of time to commence the cooking when the need arises.

**Table 3:** Use of charcoal pot types.

Type of charcoal pots	Closed air inlet	27 (6.7)
	Open-air inlet	125 (31.1)
	Open base	250 (62.2)
	Total	402 (100)
Frequency of accidents occurrence	Often	8 (2)
	Occasionally	102 (25.4)
	Never	289 (71.9)
	Total	399 (99.3)
Reasons for accidents occurrence	Mistakes	82 (74.5)
	Carelessness	28 (25.5)
Recommendation for the use of Charcoal	Yes	241 (60)
	No	149 (37)

Bracketed figures represent the percentages of each figure (frequencies) beside them.

### Analysis of variance, ANOVA in the characteristics of Zone 1, Zone 2, and Zone 3

The analysis of variance in the datasets collated from the three zones (Zones 1, 2 and 3) is presented. The statistical assumption adopted in this study is that, at the probability value less than or equal to 0.05, there are significant and otherwise no significant differences. Therefore the ANOVA analysis conducted on the socio-demographic profile of the respondents gave p = 0.032, cooking attributes of the respondents, p = 0.028, and use of charcoal pot types by the respondents, p = 0.039. Examining the p-values for all the three indices, each is far below the statistical benchmark of p ≤ 0.05. The results imply that at a 95% confidence level, there were significant differences among the demographics of the respondents in the three zones. The same applies to the cooking attributes of the respondents in the three zones. And also, the same with the use of charcoal pots by the respondents in the three zones. These differences may be attributed to the respondents' literacy and understanding levels of the importance attached to safety by the respondents of the zones, respectively. Another reason for these significant differences could be the various student-related activities, marketing and transportation, and farming activities. These affect the type of physiological body build and their cooking styles, respectively.

### Emissions from common ignition aiders

**Figure 3:**
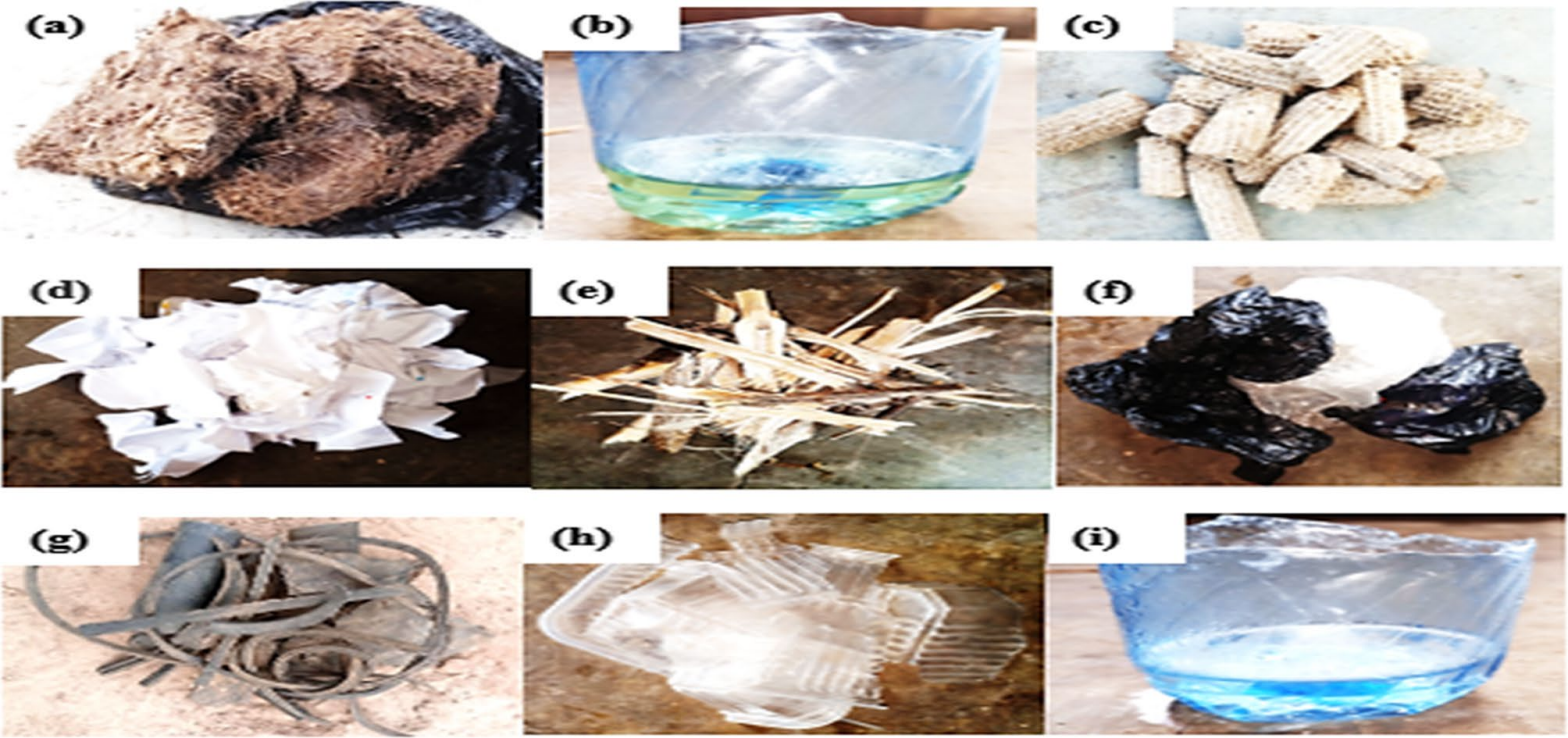
Ignition aiders (**a)** Oguso, (**b)** petrol, (**c)** maize cob, (**d)** paper (printed-on type), (**e)** wood, (**f)** nylon, (**g)** rubber, (**h)** plastic, (**i)** kerosene ( source: present study, 2022).

**Figure 4:**
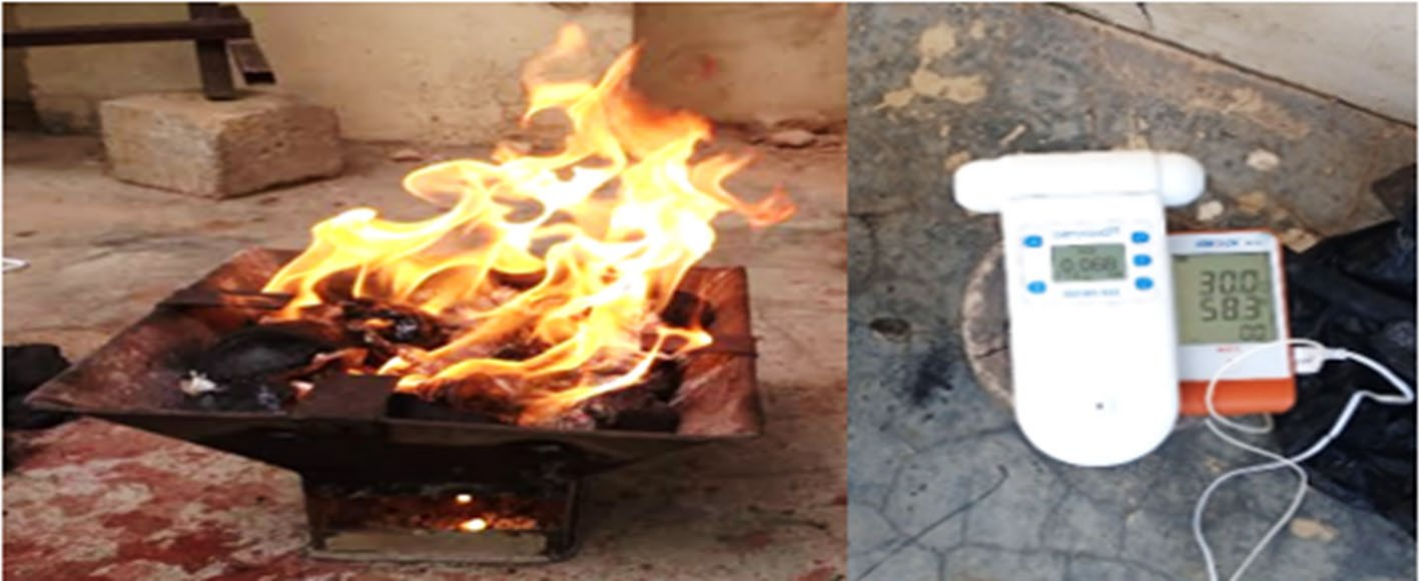
Measurement of combustion indices from a typically loaded charcoal pot ( source: present study, 2022).

Using at least one ignition aider cannot be overemphasised when any form of Charcoal is to be ignited for any purpose. This study has investigated the effects of common ignition aiders with particular reference to their major combustion indices, especially their various emissions. Therefore, Table 4 presents the analysis of emissions from the selected ignition aiders such as paper (printed-on type), nylon, rubber, plastics, petrol, *oguso* (residue of processed palm oil commonly used in south-western Nigeria), maise cob, wood, and kerosene. The typical image of the ignition aiders is presented in Figs. 3, and 4 shows how combustion indices were measured from a typically loaded charcoal pot.

**Table 4:** Common ignition aiders.

		CO (ppm)	${\mathrm{NO}}_{2}$ (ppm)	O_3 (_ppm)	VOC (ppm)	Ratio of CO/VOC
Ignition aiders (present study, 2022)	*Oguso*	186.15	1.31	0.03	6493.53	0.029
	Petrol	133.07	0.22	0.10	1115.25	0.119
	Maise cob	177.67	0.19	0.00	2713.31	0.065
	Paper	28.10	0.01	0.00	3434.54	0.008
	Wood	153.86	0.00	0.00	69,246.41	0.002
	Nylon	149.86	0.00	0.00	2532.73	0.059
	Rubber	79.42	0.00	0.00	20,942.30	0.004
	Plastic	40.42	0.00	0.01	11,665.15	0.003
	Kerosene	130.29	0.22	0.01	1777.47	0.073
Standards	ACGIH (1991) ^31^	≤ 30	≤ 3 ≤ 5	≤ 0.3	–	
	NIOSH (1992) ^32^	≤ 35	–	–	–	
	NIOSH (1988) ^33^	–	≤ 1	≤ 0.1	–	
	NJDHSS (2000) ^34^	–	≤ 1	–	–	
	OSHA (2000) ^35^	≤ 50	≤ 5	≤ 0.1 ≤ 0.3	–	
	EPA (2018) ^36^	–	–	≤ 0.07	–	
	EPA (Watson, 2021) ^37^	–	–	–	≤ 15

The values for CO content reported for all the ignition aiders investigated are significantly higher, except for paper. From Table 4, it is observable that *oguso* with 186.15 ppm has the highest value of CO content, unlike paper, 28.10 ppm, which recorded the least value. However, the ignition aider used produced higher values of CO, which are harmful to users compared with the required standards (Table 4). Variations in each value could be attributed to the chemical compositions of the respective ignition aiders. It is reported that low CO levels are an indication that an efficient combustion process is attainable^38^. Thus, this will give room for a drastic reduction of health risks and challenges associated with ignition aiders that have CO levels beyond the permissible exposure level of 30–50 ppm for an 8-h time-weighted average concentration^31,32,35^. Considering the part trace of the CO compound in each of the studied ignition aiders, the variations could be improved upon to have reduced content of the elements. Therefore, blending especially the ones with high and low values, is recommended. For, ${\mathrm{NO}}_{2}$, the recommended airborne Permissible Exposure Limit (PEL) is 5ppm^34^, 1ppm^33,34^, and an average of 3 ppm over an 8-h work shift and 5 ppm as a Short Term Exposure Limit (STEL) (AGGIH) according to New Jersey Department of Health and Senior Services^34^.

Consequently, all the ignition aiders produced ${\mathrm{NO}}_{2}$, values lesser than the recommended values except the O*guso,* which has a higher value than the NIOSH Standard^32^ (Table 4). This inference shows that any Ignition aiders are suitable, considering ${\mathrm{NO}}_{2}$ only. Also, the Occupational Safety and Health Administration (OSHA)^35^, American Conference of Governmental Industrial Hygienists (ACGIH)^31^ and Environmental Protection Agency (EPA)^36^ recommended PEL of 0.1 ppm Time Weighted Average (TWA)/0.3 ppm STEL, ≤ 0.3 ppm and ≤ 0.07 ppm TWA for ozone, respectively NIOSH^33^. All the ozone values from all the ignition aiders are within the recommended STEL/TWA except the value of petrol which is the same as the TWA value (Table 4). Volatile organic compounds, VOCs are gases (both compounds and constituents) emanating from the combustion of Charcoal and those of the various ignition aiders. High base levels of VOCs above 15 ppm, can indicate a general lack of cleanliness in space and improper ventilation^37^. However, all of the aiders produced a high quantity of VOC which is one of the severe risks experienced due to the use of Charcoal and allied (Table 4). Also, they pose various health hazards that could have short or long-term adverse effects. VOCs and particulate matter (soot) cause many long-term physical ailments, informally known as “hut lung”. This general term refers to respiratory diseases like tuberculosis, respiratory infections, and cancer. According to Mperejekumana et al*.*^39^, charcoal users (especially women) are affected at a higher rate as they are more exposed to emissions while going about their domestic chores. VOC generated from wood when used as an ignition aider is 69,246.41 ppm and has the highest value compared with other aiders. Petrol which is the least, seems the most suitable; however, it is not advisable for use domestically because petrol is highly flammable.

Relative to the value of carbon monoxide, CO recorded for paper when used as ignition aider, with a slightly higher value of VOC than petrol, it is still advisable for use, especially domestically. Because of the possibility of averting inferno since the paper is not as flammable as petrol, and on the other hand, it proffers means of reducing adverse health implications. The CO/VOC values ratio of each of the ignition aiders presented in Table 4 revealed that petrol has the highest magnitude, 0.119, while that of wood came least with 0.002. The ratios point to the fact that there is a need for good ventilation when the charcoal pot is in use. There could be a rise in the values of VOC, resulting in adverse health implications for the respondents. Ajayeoba et al.^40^, in their study on VOCs generated in petrol stations, substantiated that too much inhalation of VOCs is hazardous to human health. Thus, using temperature and relative humidity as the input parameters, they proposed a mathematical model (shown in Eq. 1), predicting the quantity of VOCs in the environment to know the level of exposure. Though the model was meant for petrol activities, since petrol is also a part of the common ignition aiders considered in this study, the model will be suitable for use in the study area. To determine the VOC level in a specific area because in the event the VOC in parts per million (ppm) exceeds the maximum permissible level of ≤ 15, it results in adverse health implications EPA (Watson, 2021).

1 $${\text{VOC}} = 64895.27 - 23029.73\left( {\text{A}} \right){\mkern 1mu} + 7039.14\left( {\text{B}} \right){\mkern 1mu} - 176.11\left( {{\text{AB}}} \right){\mkern 1mu} + 1004.84\left( {{\text{A}}^{2} } \right) - 67.54\left( {{\text{B}}^{2} } \right){\mkern 1mu} - {\mkern 1mu} 0.34\left( {{\text{A}}^{2} {\text{B}}} \right){\mkern 1mu} + 1.41\left( {{\text{AB}}^{2} } \right){\mkern 1mu} - 11.28\left( {{\text{A}}^{3} } \right){\mkern 1mu} + {\mkern 1mu} 0.14\left( {{\text{B}}^{3} } \right)$$

where; VOC is the volatile organic compound (ppm), A is the temperature (°C), and B is the relative humidity (%).

Fajobi et al*.*^41^, refer to a ternary plot as a triangular coordinate system that uses the edges of the triangle to depict its axes such that it corresponds to the composition of the three elements considered i.e., CO, ${\mathrm{NO}}_{2}$ and VOC. This plot required that the data were first normalised to 100% using the model in Eq.  (2).

2 $$A=100 \times \frac{B}{C}$$

where; A is the normalized value of each element, B is the value of each element and C is the sum total of the three elements.

**Figure 5:**
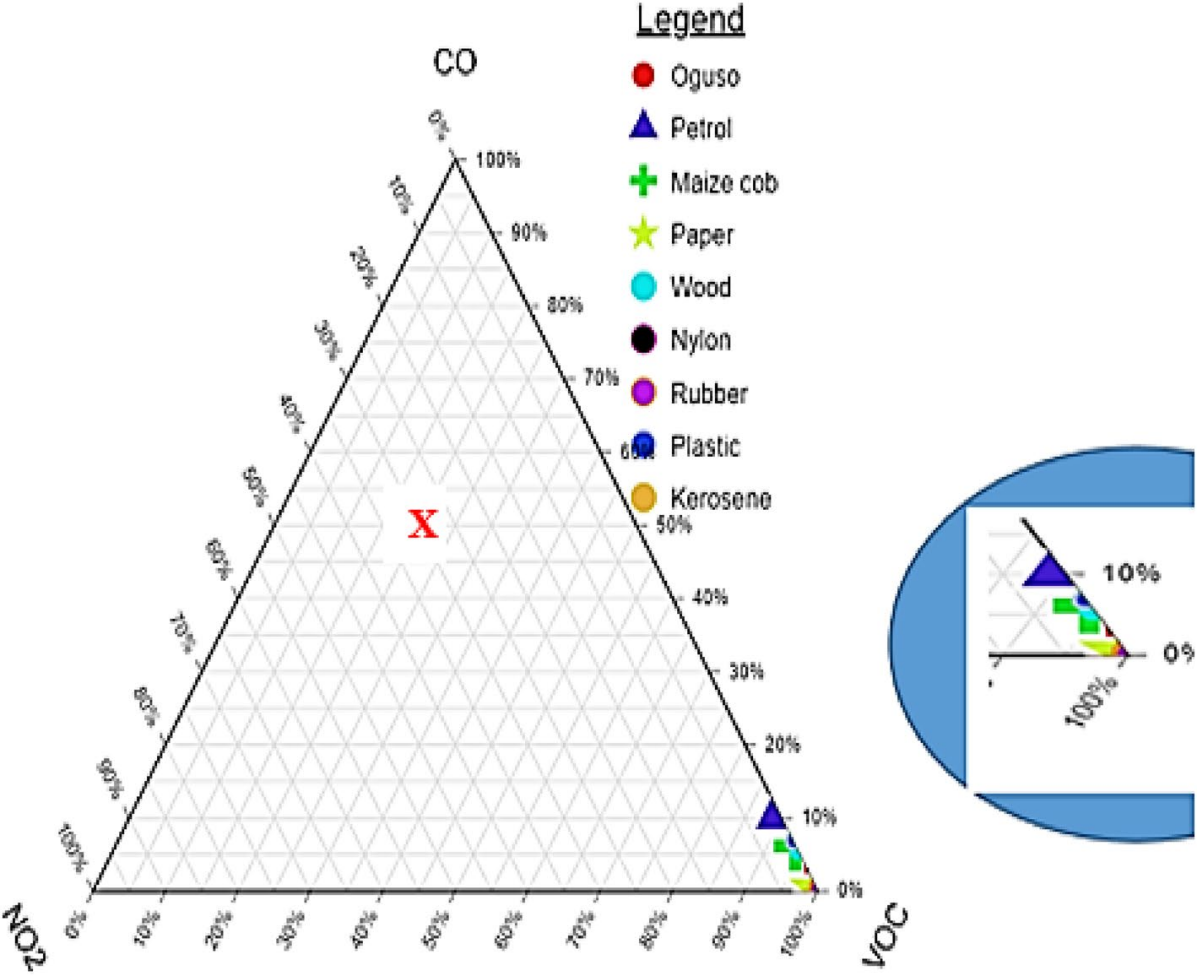
Ternary plot of combustion indices (enlarged section at the right); CO (%), ${\mathrm{NO}}_{2}$ (%) and VOC (%).

Figure 5 shows the ternary plot used to visualise and comprehend the overall variations in the combustion properties of the investigated ignition aiders. The mean values of the elements were plotted following the procedures of Vassilev et al.^42^. Three major combustion indices of the ignitions aiders were considered for nominalisation and plotted: CO, ${\mathrm{NO}}_{2}$ and VOC. An adequate examination of the plot in Fig. 5, revealed that there is an existence of high proximity among the three combustion properties even though there are variations to certain extents. The ternary plot presented in Fig. 5 determines any of the combustion indices if percentages of two out of the indices are known. Just to sum up the known two indices and subtract it from 100%. With the understanding that when combustion gases contain various compounds, the quantity of each could not be determined until an analysis is conducted. Therefore, the ternary plot is used to determine any of the compounds, especially the VOC, which requires a sophisticated instrument. Also, asides from the exorbitant cost of procurement, VOC measuring instrument demands technical know-how for effective operation. The instrument was lacking in the study area, especially with the respondents' level of education. So, the ternary plot can obtain the value of VOC at any instance once the CO, and ${\mathrm{NO}}_{2}$ values are known. For instance, at point X in Fig. 5, the percentage CO, ${\mathrm{NO}}_{2}$, and VOC are 50, 30, and 20%, respectively. The value of VOC is obtainable by adding the values of CO, and ${\mathrm{NO}}_{2}$, (i.e., 50 + 30 = 80%). Then, subtract the 80 from 100 to determine the value of VOC, which is 20%. The implication is that all the ignition aiders are viable for combustion; however, understanding the chemical content goes far in selecting the most suitable one in terms of minimal consequences on the users' health and the environment.

This study considered the details of ultimate analysis as the basis to evaluate the suitability of the ignition aiders for starting up charcoal burning. The results of the ultimate analysis are in Table 5. They are in terms of carbon, C, hydrogen, H, nitrogen, N, sulphur, S, and oxygen, O, including the calorific values of each ignition aider. The percentage carbon content ranged between 46.28 and 75.19%, hydrogen (3.35–7.50%), nitrogen (0.03–0.90%), sulphur (0.01–7.00%) and oxygen (13.59–48.25%), while the calorific values for the ignition aiders range from 17.24 to 24.94 MJ/kg. Compared with the oxygen contents, the results revealed that all the ignition aiders have high carbon contents, consistent with previous studies on the characterisation of related biomass^41^. The interpretation is a directly proportional relationship between oxygen and carbon contents. According to Fajobi et al.^41^, due to harmful and toxic gases that accompany the combustion of fuel that has much percentage of nitrogen and sulphur, they are recommended to be in low quantities in fuels. In this connection, the percentage contents of nitrogen (0.03–0.90%), sulphur (0.01–7.00%) present in the ignition aiders are acceptable.

**Table 5:** Ultimate analysis of the ignition aiders.

Ignition aiders	C (%)	H (%)	N (%)	S (%)	O (%)	CV (MJ/kg)	H/C	O/C
*Oguso*	46.28	5.59	0.90	0.10	47.13	18.26	0.12	1.02
Petrol	75.19	3.35	0.87	7.00	13.59	24.94	0.04	0.18
Maise cob	49.80	5.40	0.50	0.20	44.10	19.14	0.11	0.89
Paper	47.56	3.90	0.20	0.09	48.25	17.24	0.08	1.01
Wood	50.24	6.26	0.12	0.14	43.24	19.98	0.12	0.86
Nylon	49.70	7.50	0.30	0.01	42.49	20.84	0.15	0.85
Rubber	57.23	6.15	0.59	1.99	34.04	21.95	0.11	0.59
Plastic	62.85	5.70	0.03	0.08	31.34	23.24	0.09	0.50
Kerosene	63.04	6.35	0.16	0.09	30.36	23.83	0.10	0.48

The possibility of releasing too many toxic gases characterised by oxides of nitrogen and sulphur that can inflict Charcoal users with adverse health complications is averted. The calorific values of the ignition aiders in the range 17.24–24.94 MJ/kg are quite impressive because it matches those obtained for a similar ultimate analysis of selected biomass by Fajobi et al.^41^, although with just a slight difference. The difference could be due to the nature, sources, discrepancies in analytic processes, and chemical structure of the respective ignition aiders. The authors presumed that this result might have been positively impacted by the carbon contents of the ignition aiders, respectively. Despite all the ignition aiders, petrol has the highest magnitude of calorific value, 24.94 MJ/kg. In the opinion of this study, it should not be considered the best because it is highly susceptible to inflammability. It is also the most volatile of all the ignition aiders considered and can easily catch fire slightly but unpreparedly when a spark is about to be introduced. Considering the safety awareness of the respondents as gathered by assessments, most do not take safety as paramount in using charcoal pot; using petrol as an ignition aider may pose a threat to their lives. Therefore, although the paper has the least calorific value, 17.24 MJ/kg, its combustion indices (Table 6) are still considered the most suitable for ignition aiders for charcoal pots.

**Table 6:** Charcoals sourced from different biomass.

Charcoal	Calorific values (kJ/kg)	Author
Maise cob (*Zea* *mays*)	0.0141	Wilaipon^43^
Banana peel (*Musa* *acuminata*)	0.0189	Wilaipon^44^
Sawdust + charcoal particles	0.0249	Ajimotokan et al^45^
Soybeans (*Glycine* *max*)	12,953	Enweremadu, et al^46^
Cowpea (*Vigna* *unguiculata*)	14,372.93	Enweremadu, et al^46^
Almond shell briquette (*Prunus* *dulcis*)	19,490	Jenkins et al*.*^47^
Sawdust	20,175.81	Akowuah et al*.* ^48^
Corncob briquette (*Zea* *mays*)	20,890	Oladeji^49^
Iroko wood (*Milicia* *excelsa*)	3.2149 × ${10}^{4}$ ± 248.974	Ijagbemi et al*.*^18^
Mahogany (*Swietenia* macrophylla)	3.2230 × ${10}^{4}$ ± 337.054	Ijagbemi et al*.*^18^
Oak (*Quercus* L.)	3.2956 × ${10}^{4}$ ± 430.128	Ijagbemi et al*.*^18^
Obeche wood (*Triplochiton* *scleroxylon*)	3.3038 × ${10}^{4}$ ± 169.604	Ijagbemi et al*.*^18^
Afara wood (*Terminalia* *superba*)	3.3236 × ${10}^{4}$ ± 171.932	Ijagbemi et al*.*^18^
Suspected to be Afara or Obeche or a combination of the two	33,193.732	Present study, 2022

Bracketed words are the scientific names of the woods.

### Evaluation of energy densities of the ignition aiders

**Figure 6:**
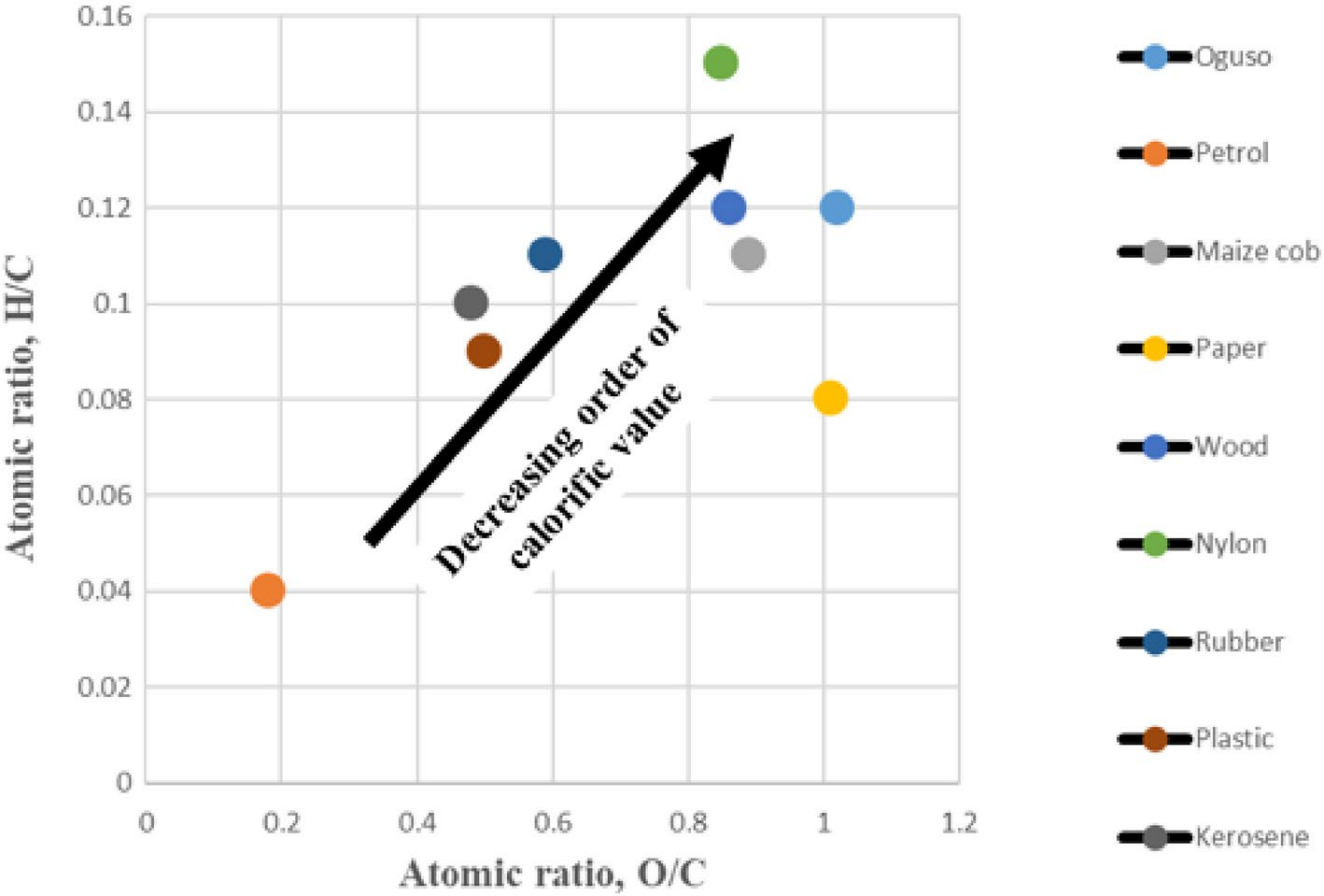
Van Krevelen plot of the ignition aiders.

A Van Krevelen plot evaluated the order of magnitude of the ignition aiders. The plot uses the classification of atomic ratios of hydrogen, oxygen, and carbon. The atomic ratios H:C and O:C (see Table 5) were plotted with the former on the vertical axis and the latter on the horizontal axis, respectively. The atomic ratio has a directly proportional relationship with energy density. The relative position of each ignition aider is located on the diagram. Compared to nylon with 0.15 H:C, which is the highest, petrol had the most negligible value of H:C (0.04). Similarly, *oguso* and kerosene had the highest, 1.02, and least, 0.18 values of O:C atomic ratios, respectively. H:C and O:C atomic ratios influenced the magnitude of calorific values (Table 5). Figure 6 revealed that petrol is the ignition aider with the densest energy, followed by paper, plastic, and kerosene. Rubber and maise cob has the same energy density, Oguso and wood also have the same energy density, while nylon is the ignition aider with the least energy density. Ignition aiders with relatively low O:C ratios exhibited more energy densities resulting in higher calorific values. Because compared to C–O bond, tremendous chemical energy is possible with C–C bonds. These results are consistent with the ratio of CO/VOC presented in Table 4, where petrol had the highest ratio. This study proposed the ratio of CO/VOC as an indicator of energy density.

This study also reviewed the energy contents of the various charcoals being used, considering their sources. The summarised review is shown in Table 6. Ajimotokan et al.^45^, produced and evaluated the calorific value of Charcoal from a blend of sawdust and charcoal particles. The calorific value obtained when subjected to bob calorimetry was 0.0249 kJ/kg. Similarly, Ijagbemi et al*.*^18^, also produced charcoal samples from oak trees, afara, obeche, mahogany, and iroko woods. The average calorific value obtained for the charcoal samples is 32,149 kJ/kg. Also, Akowuah et al*.*^48^, produced Charcoal from sawdust and the evaluated energy content of the sample was 20,175.81 kJ/kg. Oladeji^49^, Wilaipon^44^, and Wilaipon^43^, carbonised and evaluated the energy contents of Corncob briquette, Banana peel, and Maize cob, respectively. 20,890 kJ/kg, 0.0189 kJ/kg, and 0.0141 kJ/kg are the calorific values obtained from the resulting samples, respectively. Similarly, Enweremadu et al*.*^46^, investigated and established that the energy content in Charcoal obtained from cowpea and soybeans is 14,372.93 kJ/kg and 12,953 kJ/kg, respectively. While almond shell charcoal has 19,490 kJ/kg energy content, as reported by Jenkins et al*.*^47^. It could be observed from Table 5 that there are variations in the calorific values obtained from the literature compared to the one obtained for the Charcoal that was used for experimentation (33,193.732 kJ/kg). The calorific value obtained for the current study was slightly more than that of Obeche (33,038 kJ/kg) but not up to that of Afara (33,236 kJ/kg). This proximity informed the current study to presume the source of the Charcoal to be Afara or Obeche or a combination of the two. These variations could be the source of the raw wood used for Charcoal, weather condition, and carbonisation temperature, among others. Compared to others, energy contents reported for charcoals that originated from oak tree, afara, obeche, mahogany, and iroko woods by Ijagbemi et al*.*^18^, have excellent values. Thus they are recommended for use if there is a need to opt for charcoal fuel. When used alongside the ignition aider, especially paper, they promise to yield more energy values quickly.

## Conclusions and recommendations

### Conclusions

This study has established that Charcoal is widely used among the indigenes and sparingly used in zones 1 and 2 studied mostly as an alternative energy source. Charcoal requires no skills or technical know-how, but constant usage brings about expertise in the usage. The cost of procurement of other viable energy resources and poverty level are major identifiable factors that brought the use of Charcoal to the limelight. Common ignition aiders in the study area are paper, nylon, rubber, plastics, petrol, the residue of processed palm oil (locally known as *oguso* in south-western Nigeria), maise cob, wood, and kerosene. The combustion index of paper has the least value of carbon monoxide (CO) 28.1 ppm, but with a 3434.54 ppm VOC value; therefore, it is recommended for use. The average energy content reported for oak trees, afara, obeche, mahogany, and iroko woods is 32,149 kJ/kg compared to others. It is concluded that this value is excellent. When used alongside paper as an ignition aider, they are a better combination that can proffer reduced adverse health implications. Thus, considering the above facts, this study concludes that all and sundry should embrace the use of charcoal fuel for heat generation.

### Recommendations

It is recommended that charcoal fuel users should implement this study's results to avert any form of health challenges emanating from inconsistencies associated with charcoal usage. Also, regarding energy values, charcoals obtained from the carbonisation of oak trees, afara, obeche, mahogany, and iroko woods are recommended for use. The discarded papers are recommended as ignition aider when using charcoal fuel. Lastly, taking necessary safety precautions like the use of personal protective equipment and users to avoid staying long under the smoke will reduce or avert any unhealthy situation it can pose in the use of any ignition aiders.

## Data Availability

Upon request, data implemented in this study will be made available by the corresponding authors.
